# Alternatives for Uranyl Acetate Negative Staining With Respect to the Resolution Obtained After Single Particle Analysis of Erythrocruorin

**DOI:** 10.1002/jemt.24865

**Published:** 2025-03-31

**Authors:** Foteini Karapanagioti, Nicolas Cissé, Anastasiia Atamas, Artem Stetsenko, Christiaan Michiel Punter, Erica Zuidersma, Marc C. A. Stuart

**Affiliations:** ^1^ Groningen Biomolecular Sciences and Biotechnology Institute (GBB) University of Groningen Groningen the Netherlands; ^2^ Stratingh Institute for Chemistry University of Groningen Groningen the Netherlands; ^3^ Isotopes Lab, Faculty of Science and Engineering University of Groningen Groningen the Netherlands

**Keywords:** electron microscopy, image processing, lanthanide stains, negative stain, PTA, UA‐zero, uranyl acetate

## Abstract

Although cryo‐electron microscopy is widely used for determining high‐resolution structures, negative staining of proteins and supramolecular aggregates remain a valuable screening method, especially for low‐abundant proteins. The most commonly used contrasting agent is uranyl acetate (UAc). However, due to its toxicity and the strong regulations connected to the use of uranium compounds, there is an ongoing search for alternative stains, with the focus being shifted to lanthanide and tungsten compounds. In this study, the performance of neodymium acetate (NdAc), europium acetate (EuAc), ytterbium acetate (YbAc), phosphotungstic acid, and the commercially available UA‐Zero as negative staining agents for single molecule electron microscopy is evaluated. We focus not only on the observed contrast but also on the resolution that can be obtained after negative staining and single‐particle analysis. The findings demonstrate that NdAc, EuAc, and UA‐Zero can be good alternatives for the overall superior UAc. Additionally, we explore the working pH range of the stains and discuss the usability of the compounds in terms of availability and regulations.

## Graphical abstract

This study explores the efficiency of lanthanide‐based negative stains for studying single molecules with TEM, under the scope of replacing the hazardous UAc. We show that NdAc, EuAc, and UA‐Zero stains are great alternatives as they efficiently contrast protein molecules in a given range of pH and allow the 3D reconstruction of proteins at 30, 24, and 17 Å, respectively.

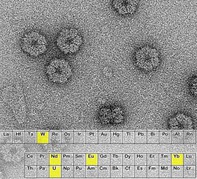

**Summary**

- Uranyl compounds for negative stains are considered to be nuclear fuel and therefore are under strict regulations.
- Lanthanides are promising alternatives for negative staining electron microscopy.
- Although uranyl acetate is the best negative staining agent, neodymium acetate, Europium acetate, and UA‐zero are good alternatives.

## 1 Introduction

Transmission electron microscopy (TEM) has been a powerful tool for observing biological specimens and inorganic materials in great detail (Orlova and Saibil 2011). However, soon after the development of electron microscopes, it became clear that the lack of heavy elements in biological samples and organic, soft materials does not provide sufficient contrast for their visualization, since the screened potential of their atoms is low, resulting in fewer scattering events (Egerton 1976; Yonekura et al. 2018). Additionally, although drying was the first and still is a widely used sample preparation method for studying self‐assembled molecules and vesicles, it poses a high risk of structural deformation and self‐aggregation of the sample, leading in many cases to the false characterization of artifacts (Hall et al. 1942; Santini et al. 1994; Schmitt et al. 1942). To circumnavigate this, reagents with high electron scattering power were used to either positively or negatively stain the sample, providing not only an increased signal‐to‐noise ratio and amplitude contrast but also partially preventing the collapse of the sample due to the extensive drying under the high vacuum conditions applied inside the electron microscope (Brenner and Horne 1959; Hall 1955; Hall et al. 1945; Horne 1991; Watson 1958).

The first negative stain images were produced by using phosphotungstic acid (PTA), which was soon followed by molybdenum and uranium stains (Hall et al. 1945; van Bruggen et al. 1960; Watson 1958). Due to the provided superb contrast and modest resolution around 15 Å, uranyl acetate (UAc) became the reagent of choice for negative staining (Boekema et al. 1995; Burgess et al. 2004; Fabre et al. 2017; Ha et al. 2016; Sawicka et al. 2016). Although most high‐resolution structural biology is nowadays performed by cryo‐electron microscopy, negative staining remains an important screening tool (Cheng and Walz 2009; Ohi et al. 2004; Rames et al. 2014). However, uranium is considered hazardous because of its chemical toxicity and radioactive properties (Keith et al. 2013).

The chemical toxicity of uranium ions and the associated health risks have been extensively documented in the past (Anthony et al. 1994; Arzuaga et al. 2015; Gao et al. 2019; Guéguen and Frerejacques 2022; Ozmen and Yurekli 1998; Vellingiri 2023). Additionally, although the uranyl compounds intended for conventional electron microscopy consist of depleted uranium—primarily containing U^238^ with typically 0.1%–0.4% U^235^—their use still carries a high risk due to internal radiochemical exposure, particularly through inhalation or ingestion (Miller et al. 2002, 2017; Monleau et al. 2006; Priest 2001). As a result, and because uranium compounds are considered “nuclear fuel,” their acquisition, transportation, storage, usage, and disposal are strictly regulated (Bleise et al. 2003; IAEA 2014, 2018; Keith et al. 2013). These restrictions turn scientists to other alternatives, such as negative stains containing lanthanides (Hosogi et al. 2015; Ikeda et al. 2011; Inaga et al. 2007; Ishii 2022; Kuipers and Giepmans 2020; Nakakoshi et al. 2011; Pinto et al. 2021; Santhana Raj et al. 2023; Sato et al. 2008; Yamaguchi et al. 2010).

Lanthanides are nonhazardous reagents with a sufficiently high atomic number providing mainly elastic electron scattering (Egerton 1976; Lambert and Ledrich 2024). The value of those reagents as effective negative stains has been described in terms of contrast compared to UAc, and it has been suggested that their density and inherent lanthanoid contraction contribute to it (Hosogi et al. 2015; Ishii 2022; Kuipers and Giepmans 2020; Nakakoshi et al. 2011). Interestingly, there have been cases such as that of ytterbium acetate (YbAc), which has been evaluated as a good negative stain for imaging viral structures but a poor one for imaging single molecules (Hosogi et al. 2015; Ishii 2022). To this date, the mechanism that drives these results remains unknown, while the value of the stains in single molecule image analysis and obtained resolution is yet to be determined.

Here, the lanthanide stains europium acetate (EuAc), neodymium acetate (NdAc), and YbAc as alternatives to UAc for negative staining of single molecules are evaluated, with respect to the resolution that can be achieved after single particle analysis. We also include in our evaluation the historically used PTA and the commercially available UA‐Zero, a stain in which ytterbium and tungsten are included and has provided promising results in imaging cross‐sections and liposomes (Pinto et al. 2021; Santhana Raj et al. 2023; Stuart 2024). The well‐studied and easily available earthworm's hemoglobin is employed as a model. We showcase the effect of the pH on the solubility of each stain, the background homogeneity and contrast on the micrographs, and discuss the best working range for each one of them. Further on, image analysis is utilized to compare the ratio of top‐to‐tilted views of the protein sample between the different stains. Finally, we attempt a 3D reconstruction of the molecule and speculate the connection between the achieved resolution and the physical properties of the stains.

## 2 Results

**FIGURE 1:**
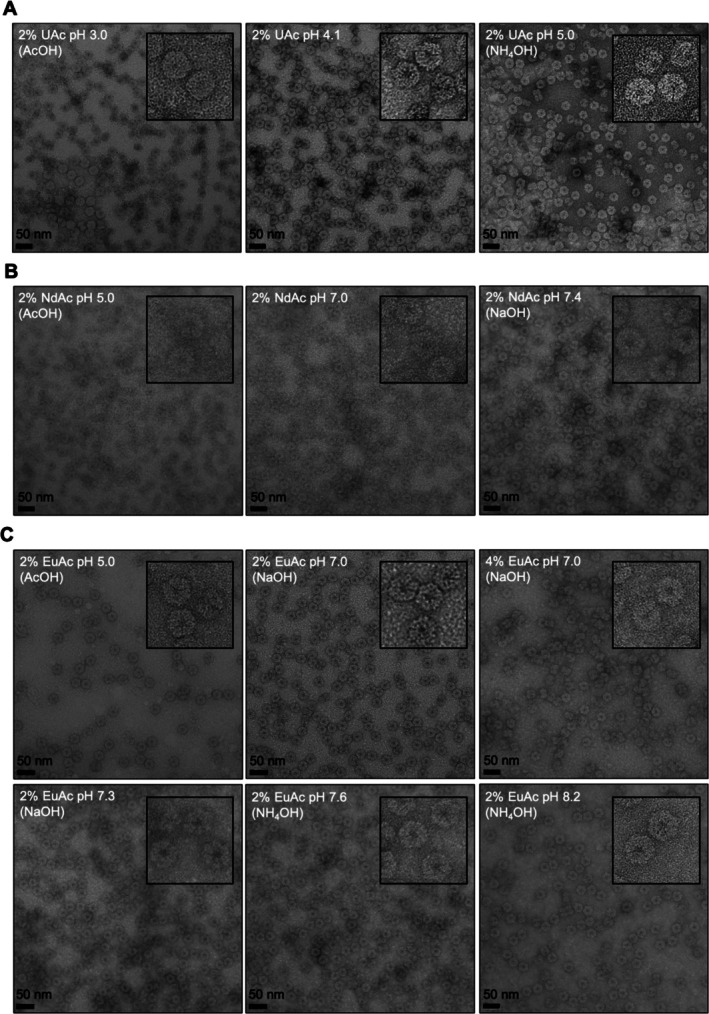
Representative micrographs of erythrocruorin stained with different solutions in a range of pH values. (A) 2% UAc stain. (B) 2% NdAc stain. (C) 2% and 4% EuAc stain. The acid or base used to reach the indicated pH value is presented in parentheses. A zoom‐in of representative molecules is shown in an inset for each micrograph.

The stains 2% UAc, 2% NdAc, 2% YbAc, 2%, and 4% EuAc were initially assessed based on the pH range of their aqueous solutions. It became apparent that the solubility of each stain in a given pH range was dependent on the nature of the acids/bases used to adjust the pH value (Table 1). Interestingly, the effect of the pH for each stain also became evident by visual observation of the related micrographs of stained erythrocruorin, the earthworm's *Lumbricus terrestris* hemoglobin (Figure 1).

**TABLE 1:** List of the different stains accessed and the respective pH range of their aqueous solutions.

Element	Stain (abbreviation)	Tested concentration (w/v %)	Starting pH	pH‐range of the soluble substance	Acid or Base used for the pH regulation below or above the starting point, respectively
^60^Nd	Neodymium Acetate (NdAc)	2%	7.0	5.0–7.4	CH_3_COOH, NaOH
^63^Eu	Europium Acetate (EuAc)	2%	5.7	5.0–7.3	CH_3_COOH, NaOH
		2%	5.7	5.0–8.2	CH_3_COOH, NH_4_OH
		4%	5.7	5.7–7.0	CH_3_COOH, NaOH
^70^Yb	Ytterbium Acetate (YbAc)	2%	6.4	6.4–7.9	CH_3_COOH, NaOH
^92^U	Uranyl Acetate (UAc)	2%	4.1	3.0–5.7	CH_3_COOH, NH_4_OH
^74^W	Phosphotungstic acid (PTA)	2%	7.0	Not studied	Not studied
^70^Yb ^74^W	UA‐Zero	Commercially available (pH 4.6–4.8)			

*Note:* The 2% PTA and UA‐Zero staining solutions were not studied in a pH range.

The pH of a 2% UAc stock solution (pH 4.1) could be decreased by the addition of acetic acid, which caused a discoloration of the initially yellow transparent solution (Figure S1A). Attempts to increase the pH of the stock solution by the addition of NaOH or (NH_4_)_2_CO_3_ resulted in immediate precipitation due to the formation of insoluble UAc sodium salts or insoluble uranyl carbonates, respectively. However, the solubility range was increased up to pH 5.7 when NH_4_OH was used as the base. In all tested conditions, the protein molecules were clearly contrasted by UAc (Figure 1A), regardless of the expected inter‐grid variability. At pH 5, the background of the micrographs lost its uniform aspect, suggesting that a liquid–liquid phase separation process took place. Indeed, certain areas of this specimen appeared rich in uranium (high contrast) in regard to others (low contrast). This coincides with the precipitation of the stain observed at high pH (Figure S1A) and suggests that there is an optimal pH range outside of which the rendered solubility of the stain possibly affects the provided contrast.

In the case of 2% NdAc stain solution, the pH could be adjusted up to pH 7.4 with the addition of NaOH, while the compound precipitated above that range. Similar to UAc, the molecules were efficiently contrasted at the studied pH range, with the observation of the particles' features becoming easier at the higher pH range (Figure 1B).

In the case of 2% EuAc stain, the pH range in which the compound remained soluble was even wider. Specifically, it was extended to pH 7.3 with the addition of NaOH, whereas NH_4_OH caused precipitation only above pH 8.2. The addition of (NH_4_)_2_CO_3_ caused immediate precipitation above the pH of the stock solution (pH 5.7). The stain provided sufficient contrast for observing the protein molecules even at pH values close to precipitation (pH 7.3, NaOH) (Figure 1C). Interestingly, when the pH of a 4% EuAc stain solution stock was adjusted above pH 7.1 by the addition of NaOH, two distinct phases were formed: a self‐standing turbid hydrogel on one hand and a clear solution on the other hand, with the two phases having approximately the same volume (Figure S1B). This phase separation process could also be caused by the increasing concentration and size of coordination polymers formed in solution. Comparing the two concentrations of the EuAc stain at pH 7.0 as adjusted with NaOH, the 2% EuAc stain produced a more uniform background on the grid in regard to the 4% EuAc stain (Figure 1C).

**FIGURE 2:**
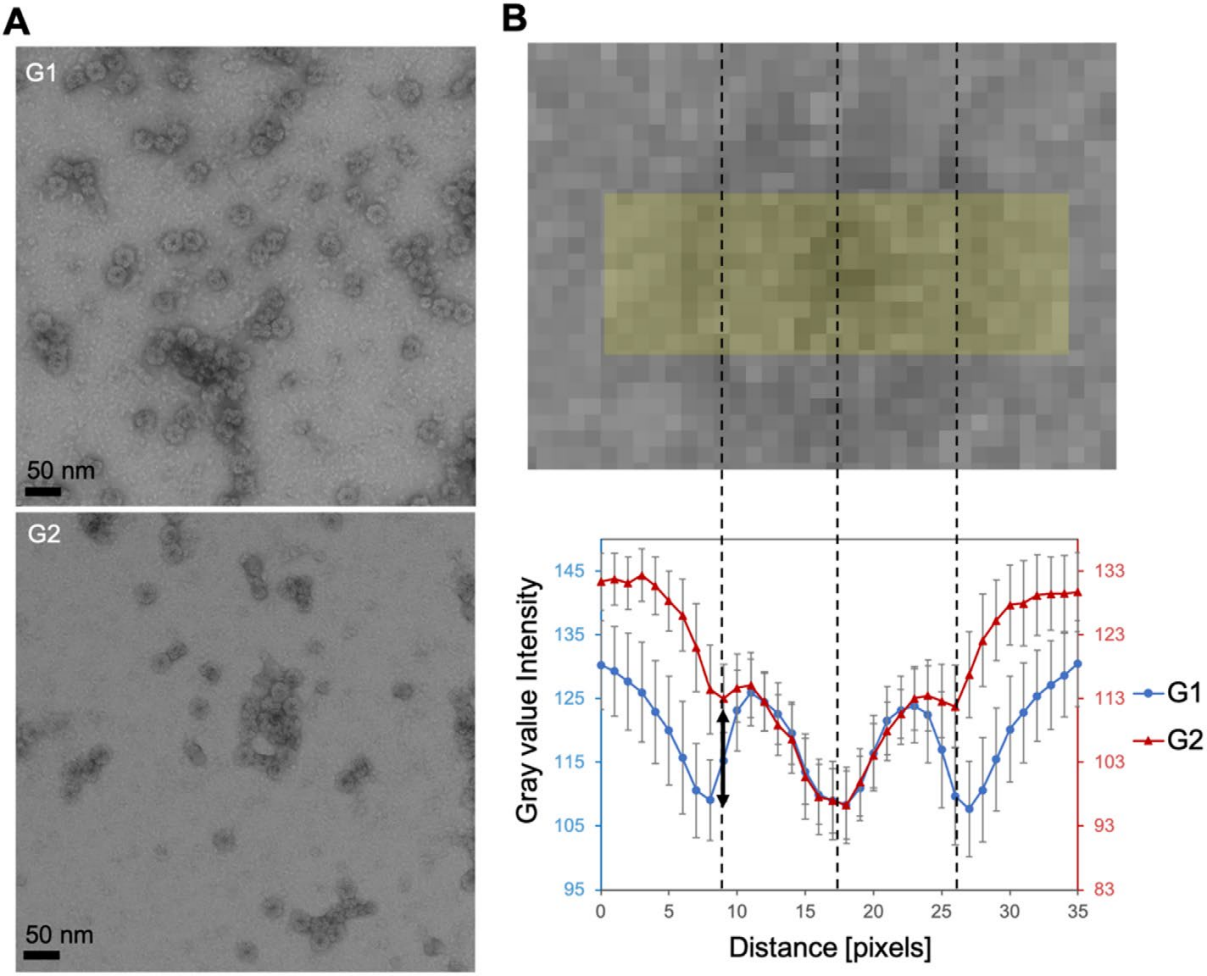
Stain assessment based on contrast. (A) Micrographs of the grids G1 and G2 imaging erythrocruorin stained with 2% UAc (pH 4.1). (B) Contrast comparison of G1 and G2 as described by the gray value intensity across particles' top views in ImageJ‐Fiji (2.14.0/1.54f) (Schindelin et al. 2012). Error bars represent the SD (*n* = 15). A representative particle selection line of 35‐pixel length and 10‐pixel width is shown above the graph. The middle of the line (pixel 17) is aligned with the middle of the molecule, while pixels 9 and 26 are aligned with the negatively stained outer rim of the molecule (dashed lines).

The use of contrast as the sole aspect for the comparison and evaluation of different stains can result in limited or even falsely interpreted results in the case of single molecules. This is showcased through the comparison of two different grids, G1 and G2, where the employed model protein was stained with 2% UAc (pH 4.1) and micrographs were acquired in focus (Figure 2A). The inter‐grid variability became apparent by visual inspection of the two micrographs. Linear selections across the top view of the molecules revealed that the difference in pixel intensity between the inner particle and its adjacent perimeter is more prominent in the case of G1 (arrow in Figure 2B). These types of defective evaluations can be outperformed by employing single‐molecule image analysis and comparing the different stains based on the obtained resolution of the molecule in question.

**FIGURE 3:**
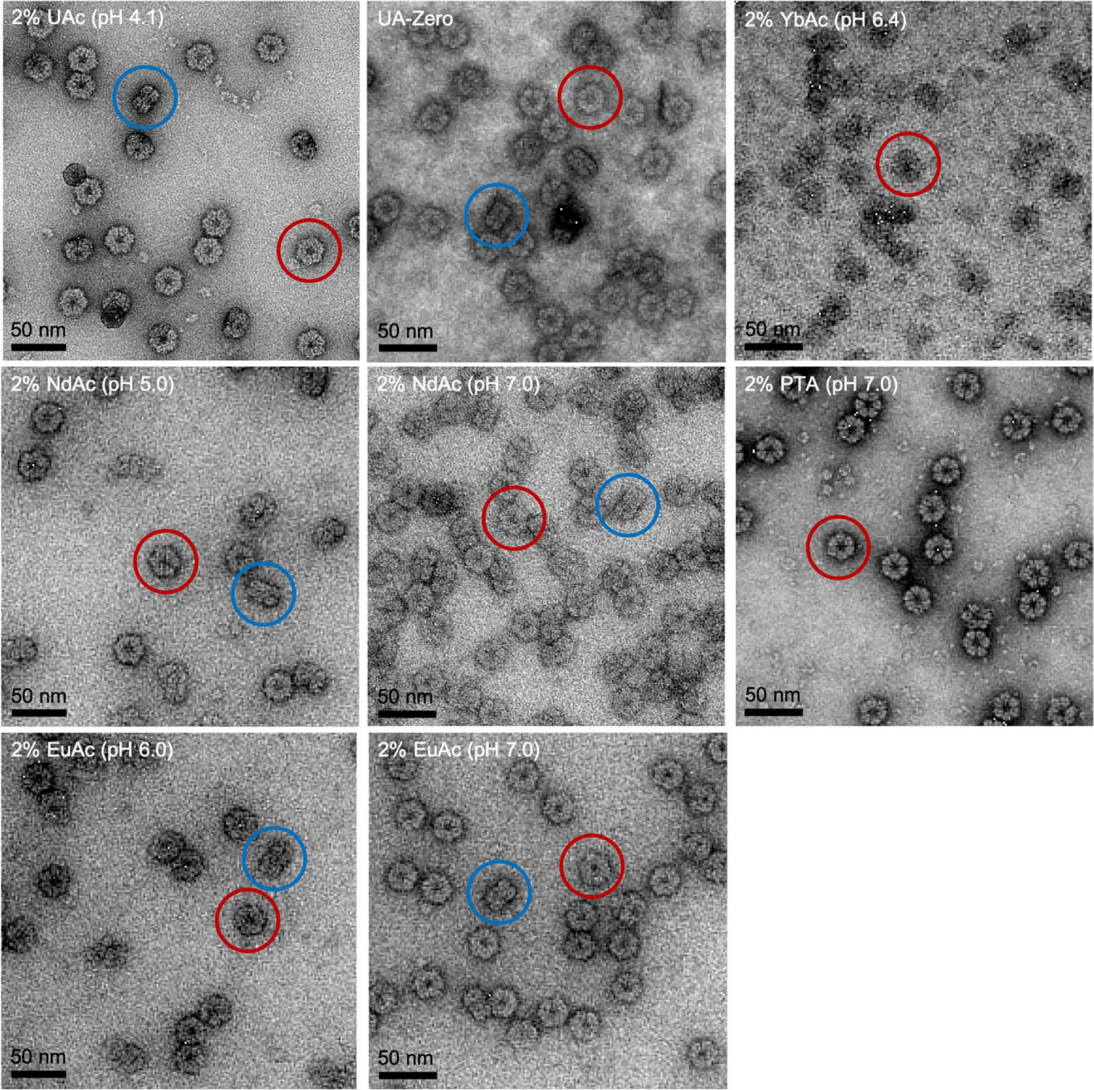
Image analysis of erythrocruorin in different stains. Representative micrographs for each accessed stain. The top and side views of the particles are indicated in red and blue circles respectively.

Further assessment of the resolution that can be obtained based on single molecule image analysis was performed for the stains 2% UAc (pH 4.1), 2% NdAc (pH 5.0 and 6.0), 2% EuAc (pH 6.0 and 7.0), 2% YbAc (pH 6.4), 2% PTA (pH 7.0), and the commercially available UA‐Zero (Figure 3 and Figure S2). Interestingly, initial observation of the acquired micrographs indicated the preferential orientation of erythrocruorin in some stains (Figure S3).

**FIGURE 4:**
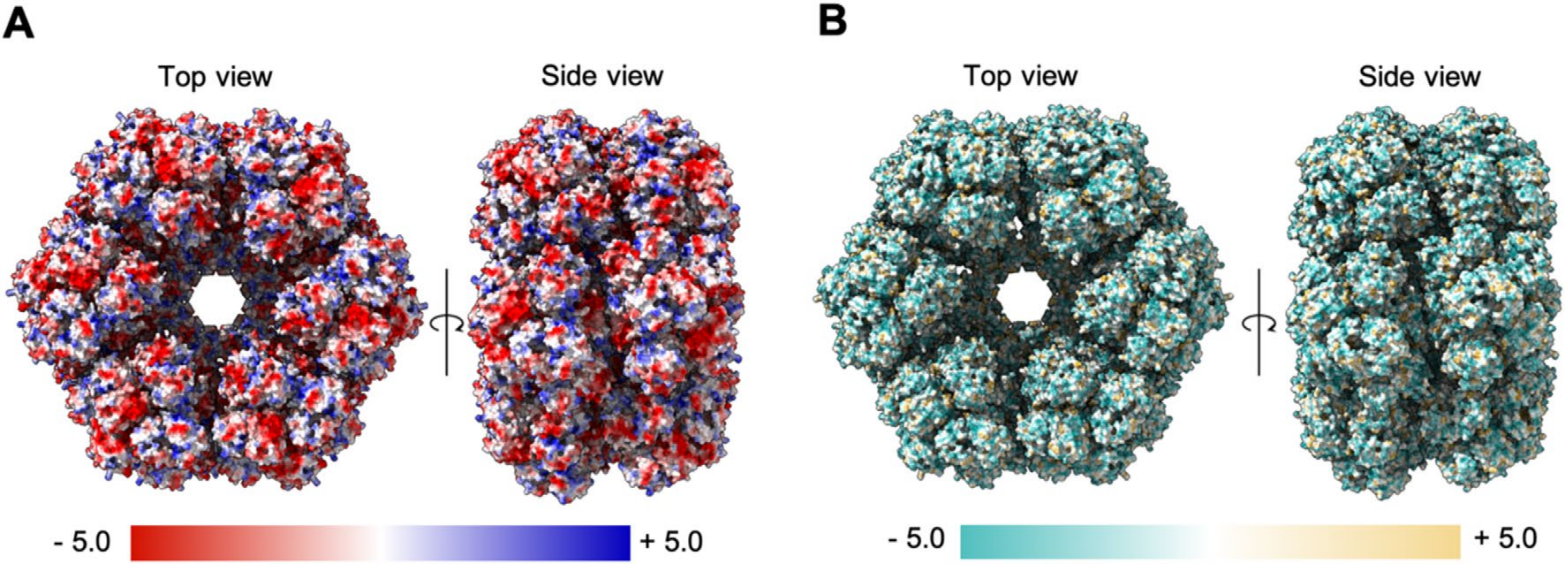
Electrostatic and Hydrophobic surface representation of erythrocruorin. (A) Electrostatic surface potential (Coulomb) of the top and side views of 2glt. Surface colors are fixed at red (−5, negatively charged) and blue (+5, positively charged). (B) Hydrophobic surface (Kyte and Doolittle) of the top and side views of 2glt. Surface colors are fixed at blue (−5, hydrophilic) and yellow (+5, hydrophobic). Visualized in ChimeraX (1.3.0) (Pettersen et al. 2021).

The hemoglobin of *L. terrestris* (PDB: 2glt) is a dodecamer of 3.6 MDa with a *D6* point‐group symmetry (Matsuda et al. 1996). Structural analysis of the protein revealed a mostly hydrophilic and slightly negatively charged surface. Analysis of the top and side views of the protein based on the degree of hydrophobicity and electrostatic interface at the surface of the molecule showed that the top and side views are quite uniform in comparison with each other when it comes to each of those characteristics (Figure 4). Thus, such inherent characteristics of the molecule could not give rise to the observed preferred orientations. A correlation, however, between the presence of preferred orientations and the atomic mass or the formation of lanthanide coordination polymers cannot be excluded.

The number of top/bottom and side/tilted molecules was determined after excluding ambiguous particles during initial 2D classifications (Figure S3). Side or tilted views of erythrocruorin were completely missing in the cases of staining with 2% YbAc (pH 6.4) and 2% PTA (pH 7.0), thus 3D reconstruction of the protein was not possible (Figure S3G,H; Table 2). Specifically, in the case of staining with 2% YbAc (pH 6.4), the particles were hardly distinguishable because of the low contrast and the grainy background on the grid, which could have contributed to the absence of distinguishable tilted views of the molecule (Figure 3 and Figure S3G). In the case of the 2% PTA (pH 7.0) stain, the protein molecules were highly contrasted, probably due to the high molecular weight of the PTA, allowing visual observation of details such as the molecules' subunits in the 2D derived classes (Figure 3 and Figure S3H).

**TABLE 2:** List of the different stains accessed with single molecule analysis.

Stain	Number of particles	Ratio of top‐to‐tilted views (2D image analysis)	Global resolution [Å] (3D image analysis)
2% NdAc (pH 5.0)	1985	1.4	30
2% NdAc (pH 7.0)	7332	1.6	30
2% EuAc (pH 6.0)	5565	3.6	24
2% EuAc (pH 7.0)	4909	5.1	27
2% UAc (pH 4.1)	12,005	1.5	15
UA‐Zero	5574	1.1	17
2% YbAc (pH 6.4)	1272	Top views only	Not applicable
2% PTA (pH 7.0)	3778	Top views only	Not applicable

*Note:* The number of particles was determined after excluding ambiguous particles during the initial 2D classifications in RELION‐3.1.4 (Scheres 2012). In the case of 2% YbAc (pH 6.4) and 2% PTA (pH 7.0), no top‐to‐tilted views ratio and resolution are reported due to the preferred top orientation of the molecule in those stains.

Despite that the commercial UA‐Zero includes both ytterbium and tungsten, the top‐to‐tilted views ratio for erythrocruorin was 1.1. Further 3D reconstruction of the molecule resulted in a global resolution of 17 Å (Table 2 and Figure S5B). On the other hand, although the top‐to‐tilted views ratio for the 2% UAc (pH 4.1) stain was calculated at 1.5, the reagent gave the highest global resolution of 15 Å (Table 2 and Figure S5A). Nonetheless, it is important to note that the number of particles that contributed to the 3D reconstruction of erythrocruorin was at least 2–6 times higher in the case of staining with 2% UAc (pH 4.1) than in other stains. When a lower number of particles was selected to match that of UA‐Zero, the obtained global resolution decreased to 18 Å as expected (Figure S4A).

A comparison of the 2% NdAc and 2% EuAc stains showed that a relatively higher resolution can be obtained with the second one, despite its higher ratio of top‐to‐tilted views indicating a high degree of preferred top orientations (Table 2). However, staining with either one of them resulted in global resolutions lower than the ones achieved with UAc and UA‐Zero, as well as a variety of top‐to‐tilted view ratios not only between the two lanthanide reagents but also between the two pH values of the same lanthanide stain (Figure S5C–F).

In the case of the low atomic mass NdAc reagent, the top‐to‐tilted ratio was close to that of 2% UAc (pH 4.1), reportedly 1.4 and 1.6 at pH 5.0 and pH 7.0, respectively. Nevertheless, it became apparent that the main representatives of the tilted views were the side ones, with the rest of them being misrepresented. Both 2% NdAc pH 5.0 and pH 7.0 resulted in a 3D reconstruction of erythrocruorin at 30 Å, despite the 3.7 magnitude difference between them regarding the number of particles (Figure S5C,D). Further clean‐up of the low‐resolution projections during subsequent 2D classifications in the case of 2% NdAc (pH 7.0) did not affect the 3D reconstruction and global resolution of the molecule, possibly indicating that the misrepresentation of low‐degree tilted views rather than the number of particles sets the resolution limitation in this case (Figure S4B).

A higher difference in the top and tilted views was observed in the case of the higher atomic mass EuAc reagent, with the prominently represented views being the top and bottom ones regardless of the applied pH. The tilted orientations were heavily misrepresented, especially in the case of the stain at pH 7.0 where the top‐to‐tilted views ratio was calculated to be 5.1. The respective ratio at the pH 6.0 stain was found to be 3.6, with clear side views of the molecule contributing to a very small extent. However, in the case of staining with 2% EuAc (pH 7.0) a higher abundance of different tilted and side orientations was observed. The highest global resolution of the molecule (24 Å) was obtained after being stained with 2% EuAc (pH 6.0) (Table 2 and Figure S5E,F).

## 3 Discussion

Negative staining has been used over the years for the characterization of single molecules, with the hazardous UAc still being at the forefront of the research as the most commonly used staining agent. However, due to uranium's toxic properties and radioactivity around 10,000 Bq g^−1^, the use of uranyl stains is increasingly restricted by stern rules regarding their purchase, transport, storage, and disposal worldwide (Bleise et al. 2003; IAEA 2014, 2018; Keith et al. 2013). These regulations have turned EM scientists to other alternatives, with the focus set on lanthanide stains.

Lanthanide compounds are not classified as hazardous, although studies have indicated some toxicity of those elements (Balaram 2019; Blinova et al. 2020; Gonzalez et al. 2014; Lambert and Ledrich 2024; Pałasz and Czekaj 2000). Thus, the usage of such stains is not as strictly regulated as it is in the case of UAc. In this study, we evaluate four lanthanide‐based stains, EuAc, NdAc, YbAc, and UA‐Zero, for negative staining of single molecules and compare them to the historically used PTA and the conventionally used UAc stains. Previous studies have discussed the contrasting nature of NdAc, YbAc, and UA‐Zero in cross sections (Hosogi et al. 2015; Kuipers and Giepmans 2020; Pinto et al. 2021; Santhana Raj et al. 2023). However, herein it is showcased that when staining single molecules, the sole use of visual contrast or brightness can be misleading. The contrast in electron microscopy arises from both scattering and phase contrast, with the latter depending strongly on the defocus during imaging according to the contrast transfer function of the microscope. Inter‐grid variability can also arise from small differences in handling, such as the amount of stain molecules evoked during sample preparation, the blotting force, or time. Thus, single particle image analysis and comparison of the obtained resolution after 3D reconstruction of the molecule are essential to evaluate these alternative negative stains.

It is apparent that each stain has a specific working pH range. At the low pH range, observation of the molecules' details becomes challenging, especially in the cases of 2% UAc (pH 3.0) and 2% NdAc (pH 5.0). Above the working pH range, precipitation or even gelation of the compound is observed. This phase separation process could be caused by the formation of increasingly large coordination polymers at increasingly high pH values. Although the polymerization of lanthanide acetates has not been described in detail, studies have shown the pH‐dependent coordination of lanthanide polymers with mono‐carboxylate ligands (Duan et al. 2010). The coordination is based on bidentate bridging, which is linked to the polymerization and ultimately hydrogelation of molecules in the presence of a mono‐carboxylate ligand, such as acetate (Azócar et al. 2014; Karraker 1969; Leong et al. 2008). An excessive degree of polymerization could potentially make the resulting coordination polymers less soluble, trigger liquid–liquid phase separation and eventually even gelation (sol–gel phase transition), as shown in the case of 4% EuAc (pH 7.1), or precipitation (solid–liquid phase separation), as shown in the case of 2% UAc (pH 6.0). The observed precipitation might also be a result of the hydrolysis of the metal due to competition between the hydroxide ions and the carboxylate ligands at a high pH (Xu et al. 2018). Even though the exact mechanism is yet to be elucidated, it becomes clear that there is an optimal degree of polymerization for each stain which depends on the pH and provides the best contrast. Our results show that the lanthanide stains remain soluble in higher pH values than UAc, even reaching up to pH 8.2 in the case of 2% EuAc when basified with NH_4_OH. Until now, the acidic nature of uranyl compounds in studies of pH‐induced conformational changes of proteins and assemblies has been overlooked because of the stain's fast fixation time (Chiang et al. 2017; Dalmau et al. 2009a, 2009b; Hu et al. 2018; Murugan et al. 2016; Velázquez‐López et al. 2018; Zhao and Craig 2003). The attribute of the lanthanide stains to retain solubility and preserve the protein structure in a basic pH range makes them great alternatives for studying such pH‐dependent changes, even though the fixation time for those stains is yet unknown.

We additionally show that EuAc and NdAc can be successfully used for the reconstruction of single molecule structures at the range of at least 24–30 Å. UAc remains superior in terms of achieved global resolution but is followed very closely by the commercial UA‐Zero (17 Å). The achieved resolution depends on multiple factors, such as the grain size of the stain, the number of particles, the plethora of different orientations that the molecule obtains upon fixation, and the uniformity of particle distributions (Aiyer et al. 2024; Baldwin et al. 2023; Baldwin and Lyumkis 2020; Gallagher et al. 2019). Interestingly, erythrocruorin shows a highly preferred top or bottom orientation when stained with 2% EuAc (pH 7.0), but the variety of well‐resolved tilted views of the molecule probably contributes strongly to the final global resolution. On the other hand, 2% YbAc (pH 6.4) cannot be considered an effective negative stain for studying protein molecules since its obtained contrast makes the particles hardly distinguishable on the grid, allowing the identification of only top or bottom particle projections. The mechanisms that drive the molecule to obtain a preferred orientation in specific stains remain to be elucidated. We suspect that multiple reasons, such as the forces posed by larger polymers in a higher pH range, the molecular weight of the stain, and a longer fixation time, could potentially drive the preferred orientation of erythrocruorin on the grid.

## 4 Conclusion

In conclusion, the lanthanide‐based stains 2% NdAc, 2% EuAc, and the commercially available UA‐Zero can be considered good staining agents for single molecule electron microscopy as an alternative to the hazardous UAc. When studying negatively stained proteins, it is important to examine if the molecules retain their expected structure in the applied pH, are efficiently contrasted, obtain a plethora of orientations, and if a 3D structure of the molecule can be reconstructed in high resolution in order to observe as much structural information as possible. The named stains sufficiently satisfy those criteria, having a broad working pH range and allowing successful reconstruction of erythrocruorin at a resolution of 24–30 Å. The UA‐Zero, despite its limited and acidic working pH range, is proven a great candidate for negatively staining protein molecules as the 3D reconstruction of erythrocruorin reaches the resolution of 17 Å with an abundance of different orientations. On the contrary, the 2% YbAc and 2% PTA stains are proved unsuitable for single molecule analysis due to the observations of inefficiently contrasted molecules and imposed preferred orientations, respectively. Ultimately, the insights obtained from this study are envisioned to serve as guidance toward the exchange of the toxic, hazardous, and heavily regulated UAc with friendlier alternatives when utilizing TEM for studying single molecules.

## 5 Experimental Section

### 5.1 Stain Preparation

Stock solutions of 2% (w/v) UAc (pH 4.1), NdAc (pH 7.0), EuAc (pH 5.7), YbAc (pH 6.4), PTA (pH 7.0), and 4% (w/v) EuAc (pH 5.7) were prepared by dissolving the respective salts in pure water. The pH was measured with a Mettler Toledo InLab Micro pH Sensor. For the acidification of the stock solutions, aliquots of CH_3_COOH were added. For the basification of the stock solutions, aliquots of either 10% aqueous NH_4_OH or aqueous NaOH were added until the salt precipitated or the solution gelled. Aliquots of CH_3_COOH were added to help dissolution when needed. The addition of (NH_4_)_2_CO_3_ resulted in the immediate precipitation of the UAc and EuAc salts. The UA‐Zero solution (Agar Scientific) was provided as a gift from van Loenen Instruments (The Netherlands) and was used as such.

### 5.2 Sample Preparation and TEM Imaging

For the sample preparation, carbon‐coated 400‐mesh copper grids were used. The grids were glow discharged for 10 s prior to sample application. In the case of the erythrocruorin sample, the material was extracted from the worm and diluted in buffer (10 mM HEPES, 100 mM NaCl, pH 7.4) before the sample preparation. Each sample (5 μL) was applied on the grid and incubated for 1 min at room temperature. Afterward, the grid was blotted with filter paper and washed with one drop of water, followed by the respective staining solution (5 μL). After 1 min, the grid was blotted and left to air dry at room temperature prior to imaging.

The samples were examined in a CM120 transmission electron microscope operated at an acceleration voltage of 120 keV with an objective aperture of 50 μm. Micrographs of different stained grids were obtained with a slow scan CCD camera (Gatan US4000) under low‐electron‐dose conditions (Oostergetel et al. 1998). For further image processing of the stained samples 2% UAc (pH 4.1), 2% NdAc (pH 5.0 and 6.0), 2% EuAc (pH 6.0 and 7.0), 2% YbAc (pH 6.4), 2% PTA (pH 7.0), and UA‐Zero, at least 50–100 micrographs were semi‐automatically recorded using the GRACE software package controlling the slow scan CCD camera on a microscope magnification of 60,000x with a physical pixel size of 3.75 Å.

### 5.3 Image Processing

Recorded micrographs of 2% UAc (pH 4.1), 2% NdAc (pH 5.0 and 6.0), 2% EuAc (pH 6.0 and 7.0), 2% YbAc (pH 6.4), 2% PTA (pH 7.0), and UA‐Zero were processed in RELION‐3.1.4 (Scheres 2012). About 1000 particles of each sample were manually picked to generate 2D references for subsequent auto‐picking. False positives or ambiguous particles belonging to low‐abundance classes were removed after 2–4 rounds of 2D classification. Splitting the 2D projections into 50–100 classes allowed the separation of the particles into two groups, namely top/bottom and tilted views, and the respective top‐to‐tilted views ratio was calculated. The samples of 2% YbAc (pH 6.4) and 2% PTA (pH 7.0) were lacking tilted views; thus, they were not further processed.

For the 3D reconstruction of erythrocruorin in the rest of the stains, the map EMD‐3434 was used as a reference, after the application of a 20 Å low‐pass filter (Afanasyev et al. 2017). No symmetry was imposed during the refinement, subsequent to which a soft mask around the density map was created. A final processing step utilizing the created mask was performed to calculate the FSC curves. The global resolution was estimated using the 0.143 cut‐off criterion with gold‐standard FSC between two independently refined half maps. For the visualization of the structures, either ChimeraX (1.3.0) or Chimera (1.15) was used (Pettersen et al. 2004, 2021).

### 5.4 Contrast Analysis

The ImageJ‐Fiji (2.14.0/1.54f) was utilized for the contrast comparison between grids (Schindelin et al. 2012). For the estimation of the gray value intensity across the particles' top views in 32‐bit images, 15 particles were selected using the line tool and utilizing a 10‐pixel width and a 35‐pixel length line.

## Author Contributions

**Foteini Karapanagioti:** data curation, conceptualization, formal analysis, investigation, methodology, project administration, visualization, writing – original draft, writing – review and editing. **Nicolas Cissé:** conceptualization, data curation, formal analysis, investigation, methodology, writing – original draft. **Anastasiia Atamas:** data curation, formal analysis, investigation, methodology. **Artem Stetsenko:** investigation, validation, visualization, formal analysis, data curation. **Christiaan Michiel Punter:** data curation, software, resources. **Erica Zuidersma:** resources. **Marc C. A. Stuart:** conceptualization, data curation, investigation, methodology, project administration, supervision, writing – original draft, writing – review and editing, funding acquisition.

## Supporting information


Data S1.

## Data Availability

The data that support the findings of this study are available from the corresponding author upon reasonable request.
