# Supplementary material for: Alternatives for Uranyl Acetate Negative Staining With Respect to the Resolution Obtained After Single Particle Analysis of Erythrocruorin
Source: Microsc Res Tech. 2025 Mar 31;88(9):2381–91. doi: 10.1002/jemt.24865 (PMC12315638; doi:10.1002/jemt.24865)
Supplement: Supplementary file 1 — Data S1. [file JEMT-88-2381-s001.docx]

# **Supporting Information**

# **Alternatives for uranyl acetate negative staining with respect to the resolution obtained after single particle analysis of erythrocruorin**

*Foteini Karapanagioti,^1^ Nicolas Cissé,^2^ Anastasiia Atamas,^1^ Artem Stetsenko,^1^ Christiaan Michiel Punter,^1^ Erica Zuidersma,^3^ Marc C.A. Stuart ^1,2^**

^1^ Groningen Biomolecular Sciences and Biotechnology Institute (GBB), University of Groningen, 9747 AG Groningen, The Netherlands

^2^ Stratingh Institute for Chemistry, University of Groningen, 9747 AG Groningen, The Netherlands

^3^ Isotopes lab, Faculty of Science and Engineering, University of Groningen, 9747 AG Groningen, The Netherlands

* Correspondence to: [m.c.a.stuart@rug.nl](mailto:m.c.a.stuart@rug.nl) (Marc C.A. Stuart)


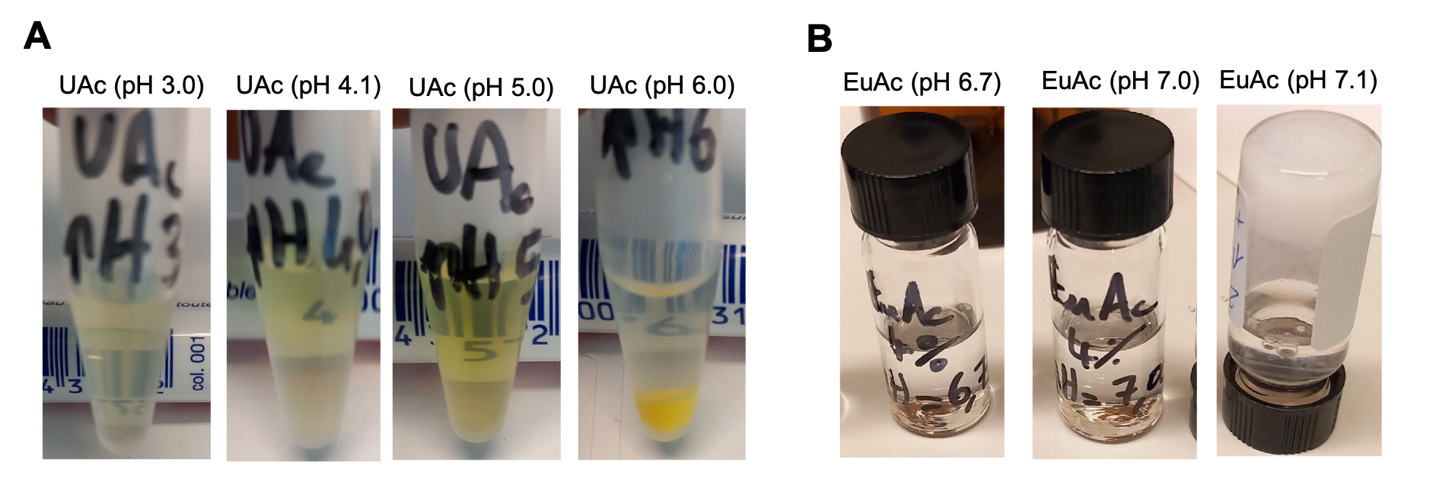


**Figure S1. Solubility pH range of UAc and EuAc stains.** (A) Different derivatives of the initial stock solution of 2% UAc pH (4.1), either acidified (with CH_3_COOH) or basified (with NH_4_OH). At pH 6.0 the stain precipitates. (B) Different derivatives of the initial stock solution of 4% EuAc pH (5.7), basified with the addition of NH_4_OH. At pH 7.1 there is a phase separation.


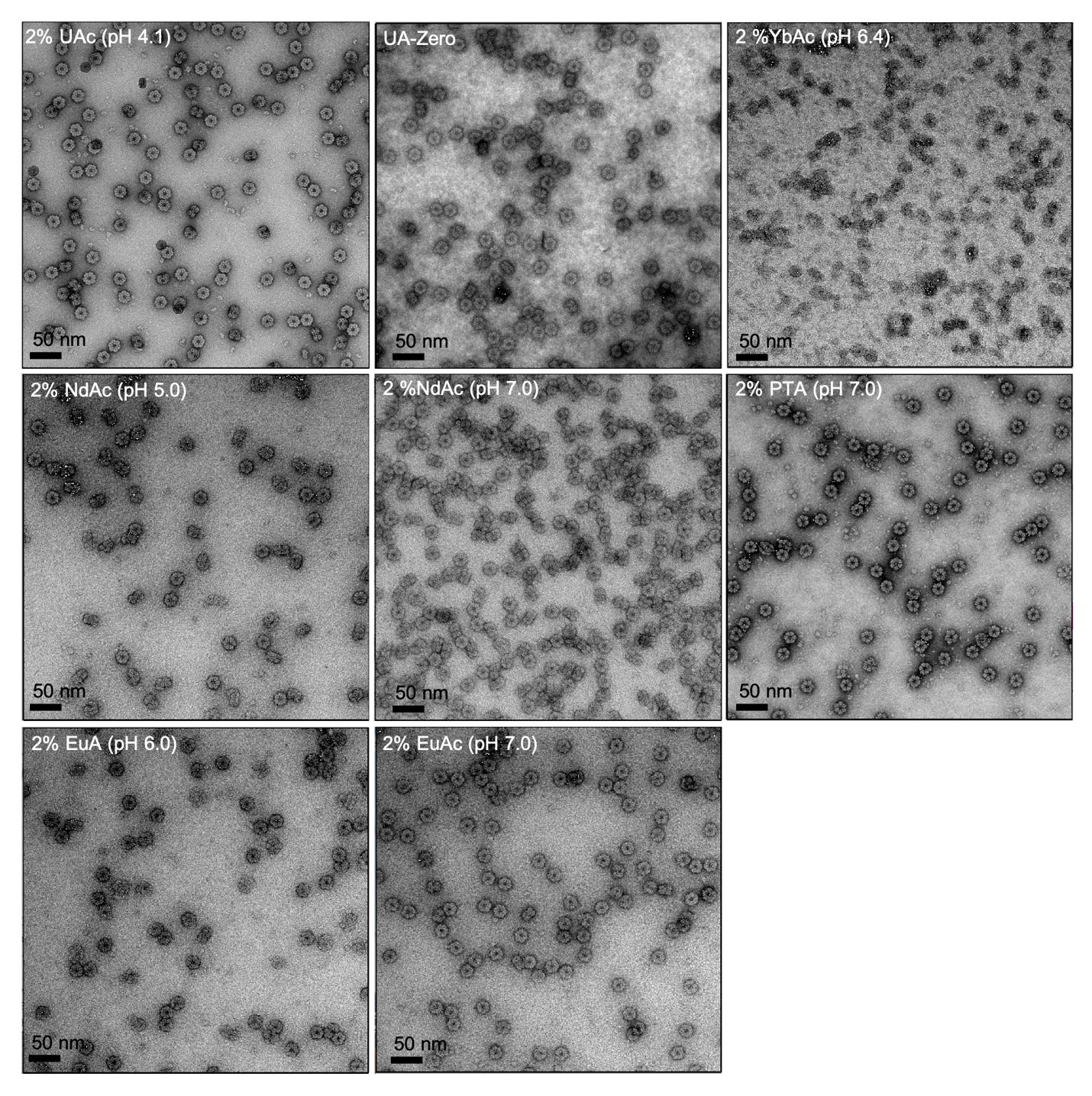


**Figure S2. Representative micrographs used for image analysis of erythrocruorin stained with different solutions.**

**
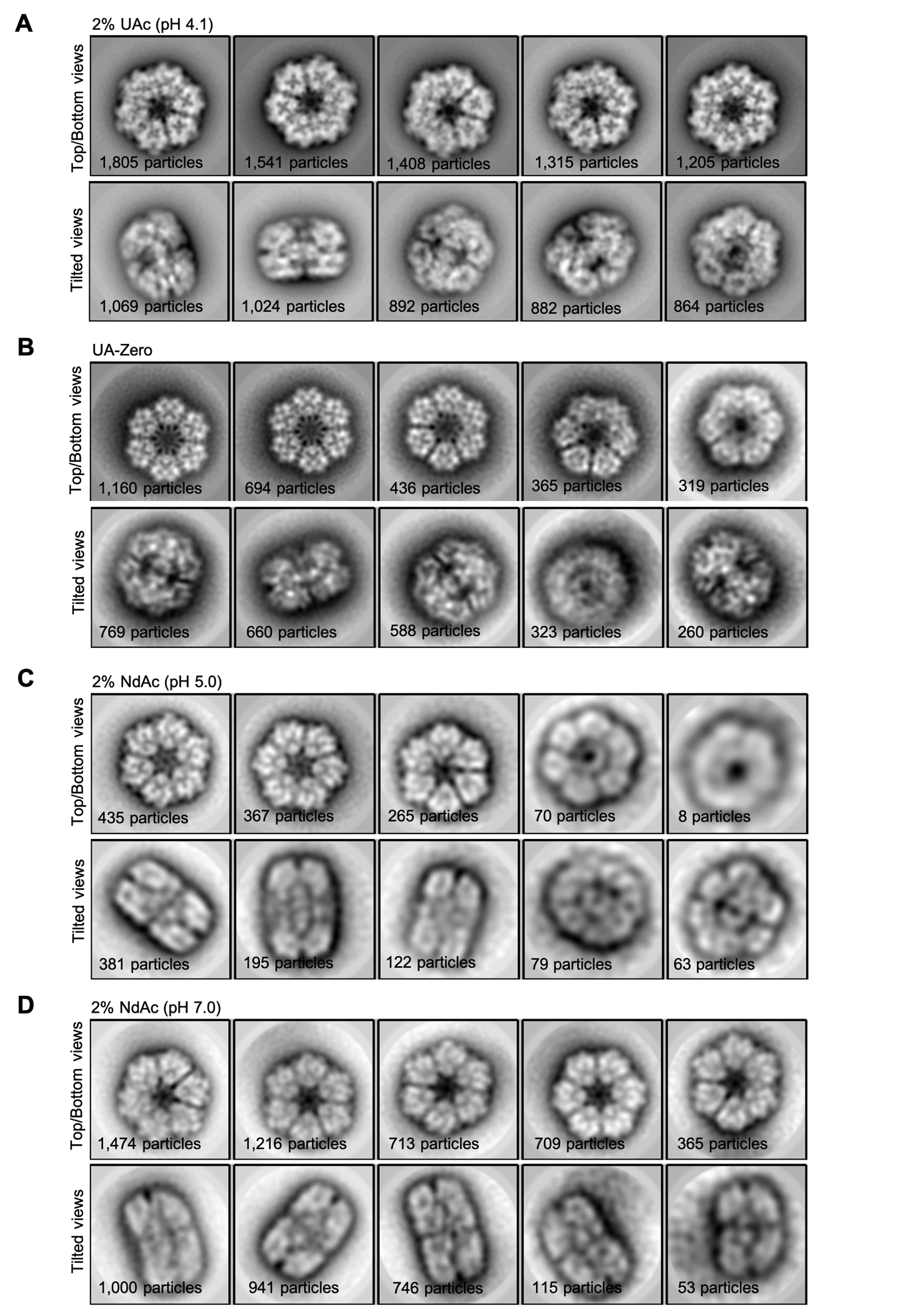
**

**
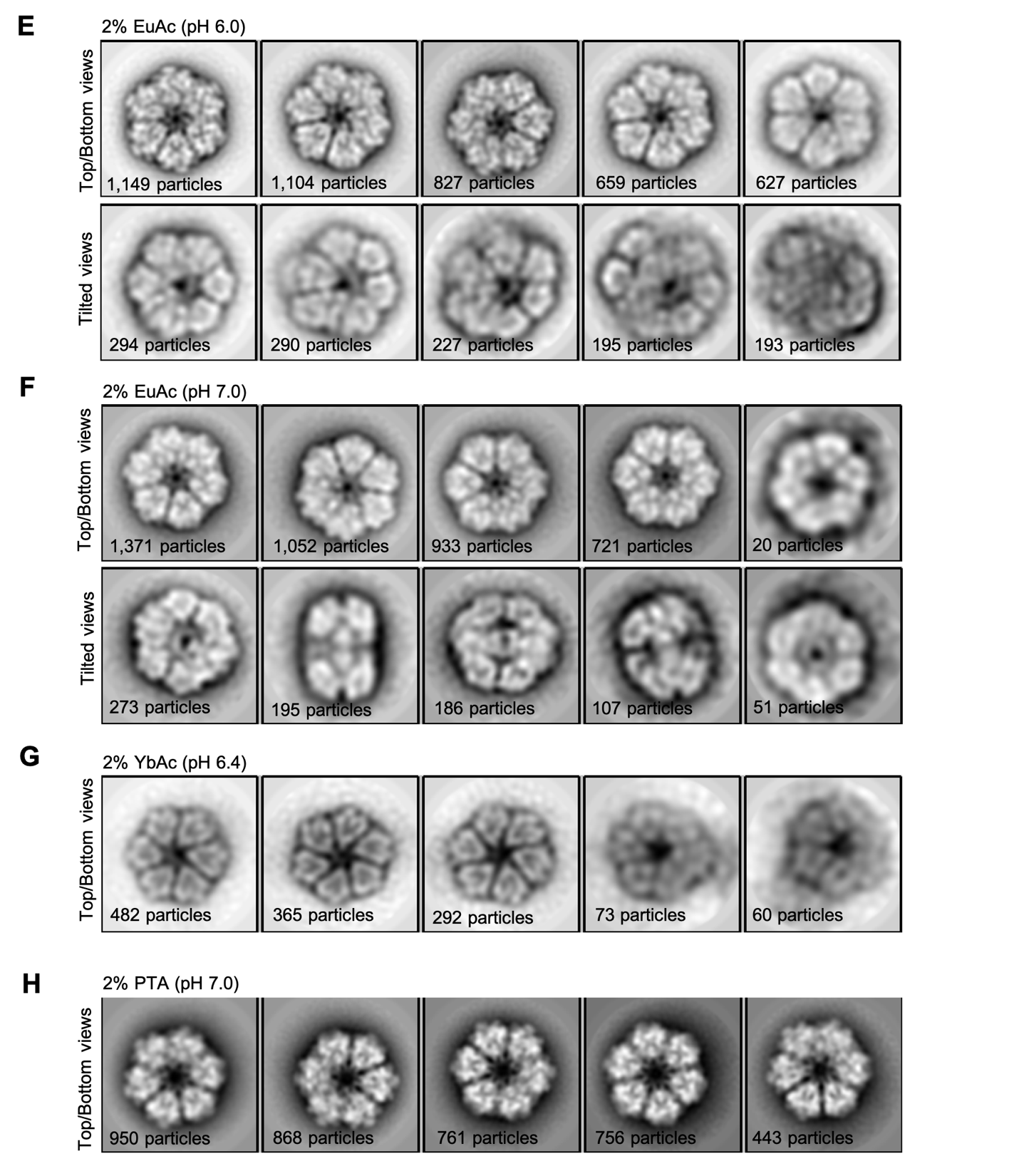
**

**Figure S3. 2D projections of erythrocruorin in different stains.** Initial splitting of the 2D projections in 50-100 classes allowed separation of the particles in two groups, top/bottom and tilted views, in each of the stains (A) 2% UAc (pH 4.1), (B) UA-Zero, (C) 2% NdAc (pH 5.0), (D) 2% NdAc (pH 7.0), (E) 2% EuAc (pH 6.0), (F) 2% EuAc (pH 7.0), (G) 2% YbAc (pH 6.4), or (H) 2% PTA (pH 7.0). The respective particles from each group were further classified in 5 classes presented here. The variations in the different orientations of the molecule in each of the stains can be observed. The number of particles contributing to each class as calculated by RELION-3.1.4 is reported at the bottom left of each projection.

**
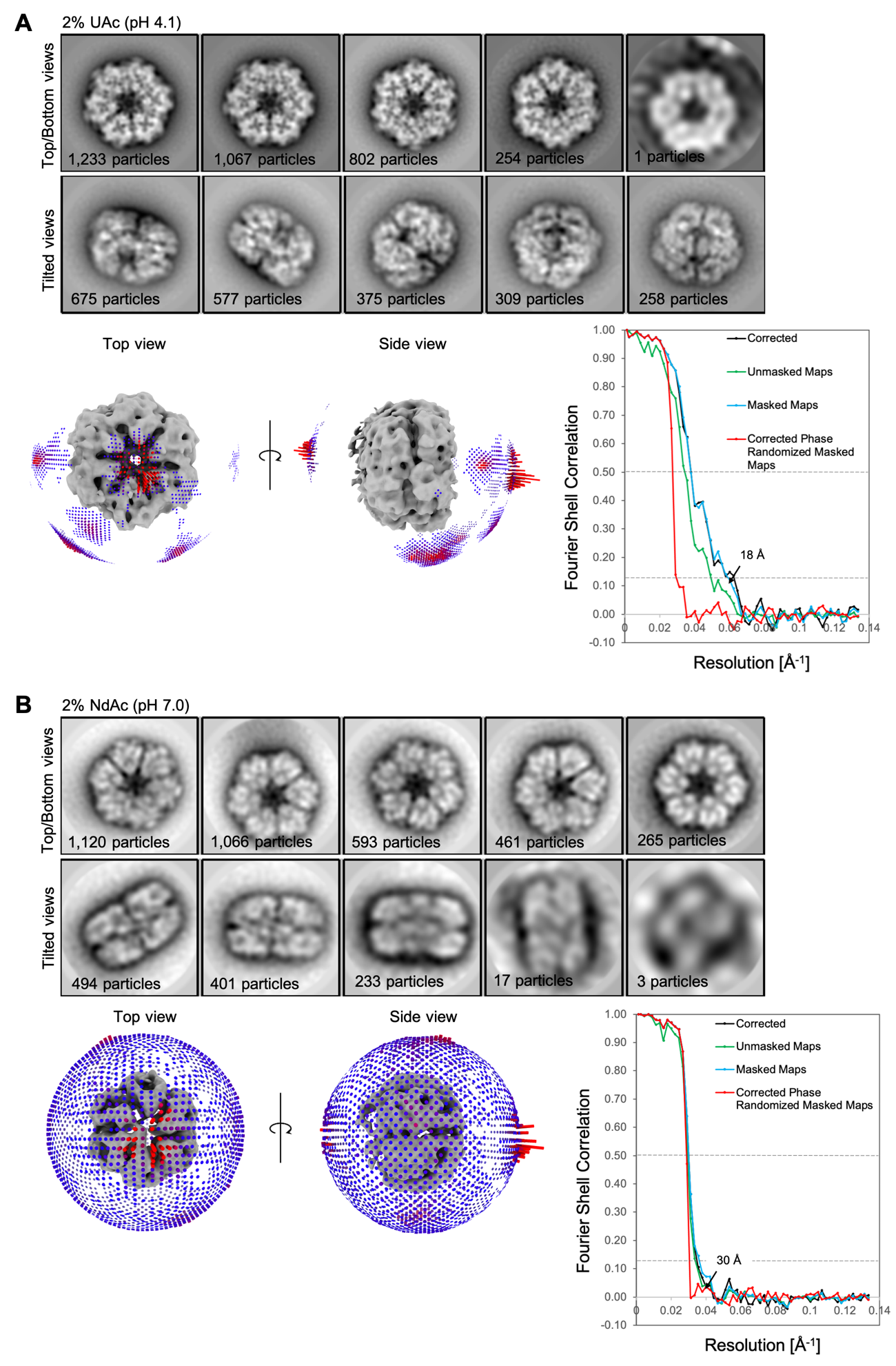
**

**Figure S4. Image analysis of erythrocruorin in 2% UAc (pH 4.1) and 2% NdAc (pH 7.0).** Several rounds of extensive 2D classifications resulted in (A) 5,551 particles in the case of 2% UAc (pH 4.1) staining and (B) 4,653 particles in the case of 2% NdAc (pH 7.0) staining. Splitting of the 2D projections in 50-100 classes allowed separation of the particles in two groups, top/bottom and tilted views. The respective particles from each group were further classified in 5 classes and are shown on the top. The variations in the different orientations of the molecule in each of the stains can be observed. The number of particles contributing to each class as calculated by RELION-3.1.4 are reported at the bottom left of each projection. The density map of the structure and angular distribution of particles that contributed to the final map of the molecule are shown on the bottom left. The height and color of the cylinder bars is proportional to the number of particles in the respective views (short and blue: low number of particles, long and red: high number of particles). The Fourier Shell Correlation (FSC) curve as a function of resolution for the final density map is shown on the bottom right. The global resolution at which the gold-standard FSC drops below the 0.143 threshold is indicated.

**
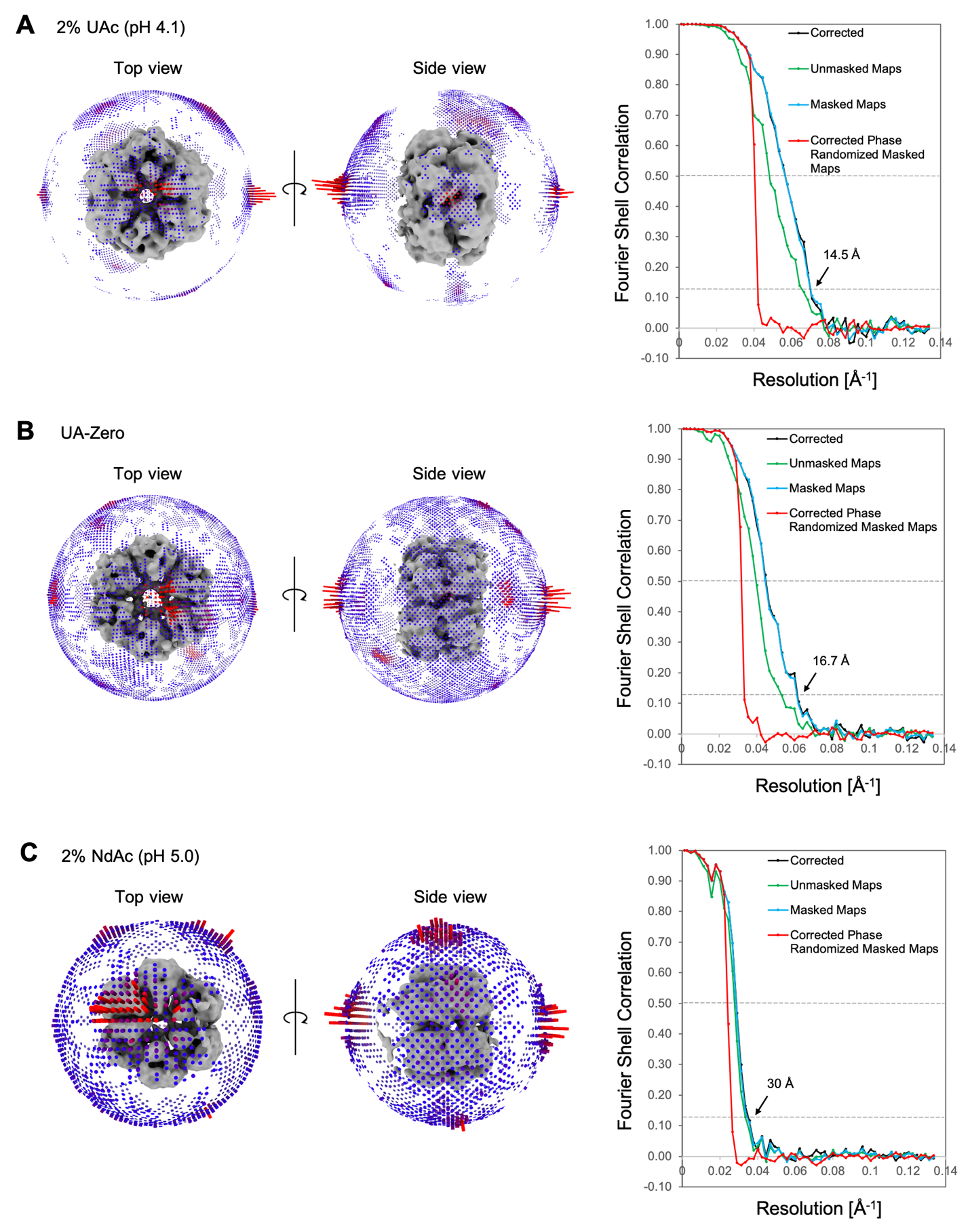
**

**
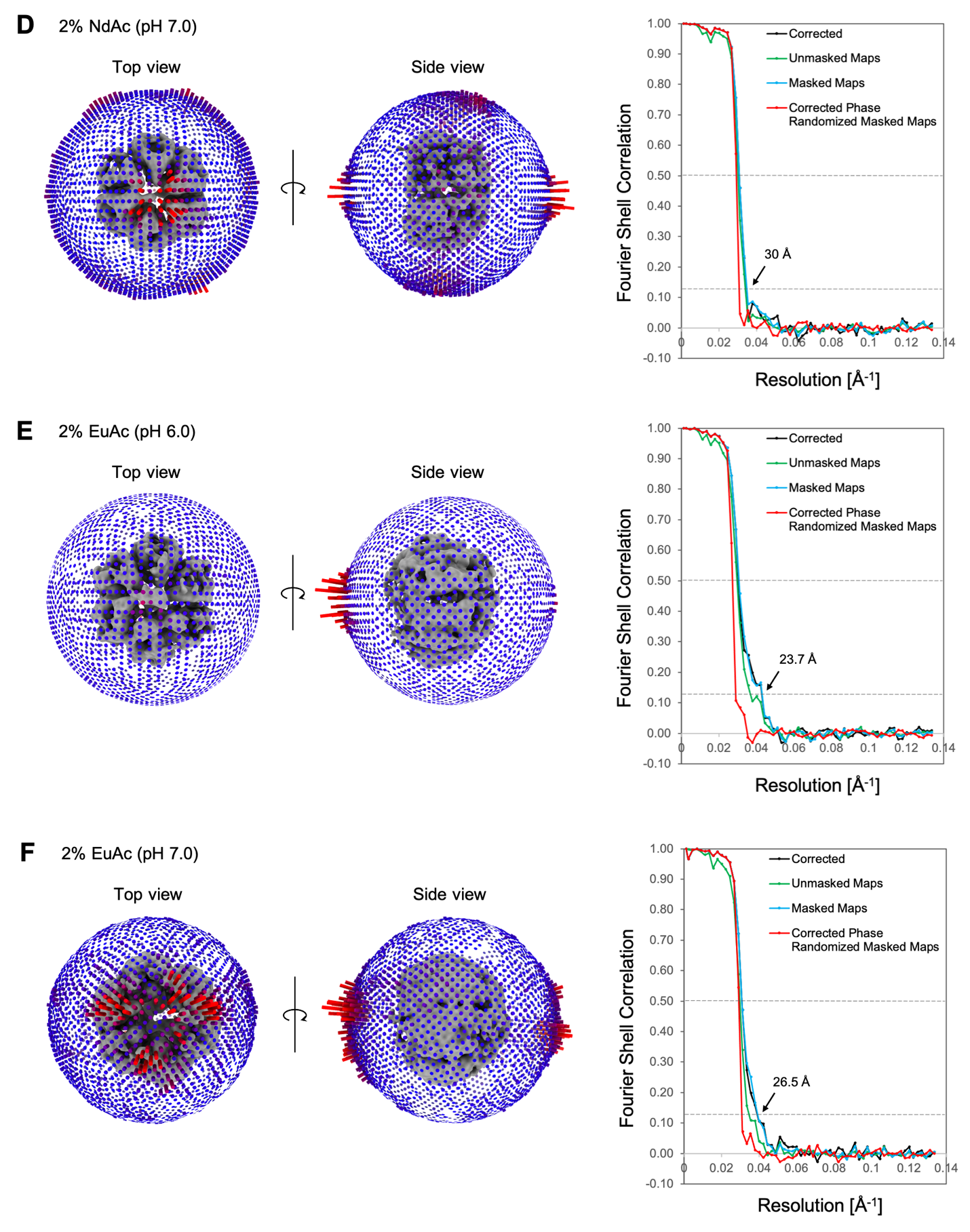
**

**Figure S5. 3D image analysis of erythrocruorin in different stains.** 3D reconstruction of erythrocruorin stained with (A) 2% UAc (pH 4.1), (B) UA-Zero, (C) 2% NdAc (pH 5.0), (D) 2% NdAc (pH 7.0), (E) 2% EuAc (pH 6.0), or (F) 2% EuAc (pH 7.0), in RELION-3.1.4. The density map of the structure and angular distribution of particles that contributed to the final map of the molecule are shown on the left. The height and color of the cylinder bars is proportional to the number of particles in the respective views (short and blue: low number of particles, long and red: high number of particles). The Fourier Shell Correlation (FSC) curve as a function of resolution for the final density map is shown on the right. The global resolution at which the gold-standard FSC drops below the 0.143 threshold is indicated.
